# Translational Application of 3D Bioprinting for Cartilage Tissue Engineering

**DOI:** 10.3390/bioengineering8100144

**Published:** 2021-10-18

**Authors:** Sophie McGivern, Halima Boutouil, Ghayadah Al-Kharusi, Suzanne Little, Nicholas J. Dunne, Tanya J. Levingstone

**Affiliations:** 1 Advanced Manufacturing Research Centre (I-Form), School of Mechanical and Manufacturing Engineering, Dublin City University, D09 NA55 Dublin, Ireland; sophie.mcgivern2@mail.dcu.ie (S.M.); halima.boutouil2@mail.dcu.ie (H.B.); ghayadah.alkharusi2@mail.dcu.ie (G.A.-K.); nicholas.dunne@dcu.ie (N.J.D.); 2 Centre for Medical Engineering Research (MEDeng), Dublin City University, D09 NA55 Dublin, Ireland; 3 Insight SFI Research Centre for Data Analytics, Dublin City University, D09 NA55 Dublin, Ireland; suzanne.little@dcu.ie; 4 Advanced Processing Technology Research Centre, Dublin City University, D09 NA55 Dublin, Ireland; 5 Biodesign Europe, Dublin City University, D09 NA55 Dublin, Ireland; 6 Trinity Centre for Biomedical Engineering (TCBE), Trinity Biomedical Sciences Institute, Trinity College Dublin, D02 PN40 Dublin, Ireland; 7 Advanced Materials and Bioengineering Research Centre (AMBER), Royal College of Surgeons in Ireland and Trinity College Dublin, D02 PN40 Dublin, Ireland; 8 School of Pharmacy, Queen’s University Belfast, 97 Lisburn Road, Belfast BT9 7BL, UK

**Keywords:** cartilage, 3D bioprinting, tissue engineering

## Abstract

Cartilage is an avascular tissue with extremely limited self-regeneration capabilities. At present, there are no existing treatments that effectively stop the deterioration of cartilage or reverse its effects; current treatments merely relieve its symptoms and surgical intervention is required when the condition aggravates. Thus, cartilage damage remains an ongoing challenge in orthopaedics with an urgent need for improved treatment options. In recent years, major advances have been made in the development of three-dimensional (3D) bioprinted constructs for cartilage repair applications. 3D bioprinting is an evolutionary additive manufacturing technique that enables the precisely controlled deposition of a combination of biomaterials, cells, and bioactive molecules, collectively known as bioink, layer-by-layer to produce constructs that simulate the structure and function of native cartilage tissue. This review provides an insight into the current developments in 3D bioprinting for cartilage tissue engineering. The bioink and construct properties required for successful application in cartilage repair applications are highlighted. Furthermore, the potential for translation of 3D bioprinted constructs to the clinic is discussed. Overall, 3D bioprinting demonstrates great potential as a novel technique for the fabrication of tissue engineered constructs for cartilage regeneration, with distinct advantages over conventional techniques.

## 1. Introduction

Articular cartilage is a smooth, wear-resistant, highly specialised hyaline cartilage found at the ends of bones within synovial joints where it reduces friction to allow smooth joint movement [1]. As a result of its avascularity and aneurality, cartilage has extremely limited self-regeneration capabilities, thus damage to the articular cartilage from pathological conditions such as osteoarthritis (OA) and rheumatoid arthritis (RA), and traumatic injury pose a significant challenge to orthopaedic surgeons. OA is the most common joint disorder in the world. Minor symptoms experienced during early-stage disease can be managed through medication and physiotherapy; however, as the disease progresses, severe articular cartilage damage occurs. OA has a significant impact on a patient’s quality of life, causing severe pain, stiffness, and swelling in the affected region. Over 300 million people globally suffer from OA as of 2019 [2], resulting in a significant economic burden [3]. The current treatments for conditions affecting the articular cartilage consist primarily of pain management medication and physiotherapy, with surgical intervention required in more severe cases. Current surgical approaches include microfracture, subchondral drilling, abrasion arthroplasty, autologous chondrocyte implantation (ACI), matrix-assisted ACI (MACI), and osteochondral autograft/allograft transplantation (OAT) [4]. While these techniques are widely applied clinically, there are associated limitations and complications such as donor site mobility, graft hypertrophy, and inconsistent repair tissue associated with them [4]. Ultimately, a total joint replacement is required for end-stage disease. Thus, the development of new approaches capable of effectively regenerating damaged cartilage tissue is imperative.

Tissue engineering, an interdisciplinary field that combines biomaterial scaffolds, cells, and signalling agents to develop biological substitutes capable of restoring, maintaining, or improving tissue function, shows promise for the development of new approaches for the repair of cartilage tissue [5]. Within the tissue engineered construct, the scaffold and signalling agents function to direct cells to produce the required tissue type, thus this approach offers advantages over standard cell-based therapies. An ideal scaffold should replicate the unique mechanical and biological properties of the native ECM of the desired tissue and have a porous structure that allows for cell attachment and nutrients exchange. Three-dimensional bioprinting, an additive manufacturing process, has recently been applied to the fabrication of tissue-engineered constructs for a range of applications including cartilage defect repair. The process involves the layer-by-layer deposition of cell-laden biomaterials, called bioinks. The 3D bioprinting technique can be applied to replicate the complex organisation of cells and ECM within native tissues due to its ability to precisely control material deposition [6]. Additionally, cells, drugs, and bioactive molecules can be incorporated in a spatially controlled manner within the constructs for an enhanced cellular response, and thus, 3D bioprinting boasts major advantages over current scaffold fabrication techniques. The selection of an appropriate bioink is a critical consideration when designing 3D bioprinted constructs. Bioinks must comply with a wide range of stringent requirements, including biocompatibility and biodegradability, while also possessing the necessary rheological properties to ensure good printability. Often, adjusting factors that improve printability such as increased viscosity, induce a harsh environment for the survival and functionality of cells. A delicate compromise between these factors is therefore required to achieve the optimal bioink and construct compositions [7]. Three-dimensional bioprinted constructs require the ideal biochemical composition, architecture, surface properties, and mechanical properties to support cell growth, proliferation, and differentiation, and to withstand the biological environment post-implantation. This review focuses on the recent advances in the development of bioinks and 3D bioprinted constructs for cartilage tissue engineering applications and discusses the potential for the translation of these constructs to the clinic for the treatment of damaged articular cartilage.

## 2. Tissue Engineering Approaches for Cartilage Tissue Engineering

Cartilage has a dense structure comprised of highly specialised cells, known as chondrocytes and chondroblasts, embedded in the cartilaginous extracellular matrix (ECM) which is comprised mainly of proteoglycans, glycoproteins, collagen fibres, elastin fibres, and water. Articular cartilage has a complex layered structure consisting of four zones: (i) a superficial zone, (ii) a transitional zone, (iii) a deep zone, and (iv) a calcified zone, each with different matrix compositions, structural organization, and cell density. The superficial zone contains collagen type II fibers aligned parallel to the cartilage surface, the transition zone contains randomly orientated collage II fibers, while the in the deep zone type II collagen fibers are arranged vertically. This unique anatomy results in gradient physical, mechanical, and biological properties which makes articular cartilage damage increasingly complex to repair and poses challenges for the design of tissue-engineered constructs for cartilage repair. 

A wide range of fabrication techniques have been used to fabricate porous scaffolds for cartilage tissue engineering applications including porogen-leaching [8], gel-pressing [9], solvent-casting [10], electrospinning [11], and freeze-drying [12,13]. More recently, approaches that enable the fabrication of layered scaffolds that more closely replicate the graduate nature of cartilage tissue have been developed [13,14]. While these techniques allow control of the material composition in each layer, spatial control over the organisation of cells and growth factors within the constructs cannot be effectively achieved. Thus, 3D bioprinting offers the potential to achieve constructs for cartilage tissue repair that more closely mimic the native tissue environment and thus hold a greater potential to achieve rapid, long-lasting repair of cartilage tissue.

## 3. 3D Bioprinting for Cartilage Tissue Engineering Applications

Three-dimensional bioprinting describes the manufacture of structures through the deposition of materials in a layer-by-layer process. These layers can be adhered together using different techniques, including heat, UV light, fusing agents, and crosslinking techniques, depending on the 3D bioprinting technique used [15]. The 3D bioprinting process allows for the production of complex porous structures and as such, has excellent potential as a technique for the fabrication of constructs for cartilage tissue engineering applications [16]. The highly controllable nature of the 3D bioprinting process enables the fabrication of constructs that replicate the layered structure of cartilage ECM due to its ability to precisely control material deposition and cell positioning. Thus, it offers major advantages over traditional fabrication techniques [17]. 

### 3.1. Types of 3D Bioprinting

**Figure 1:**
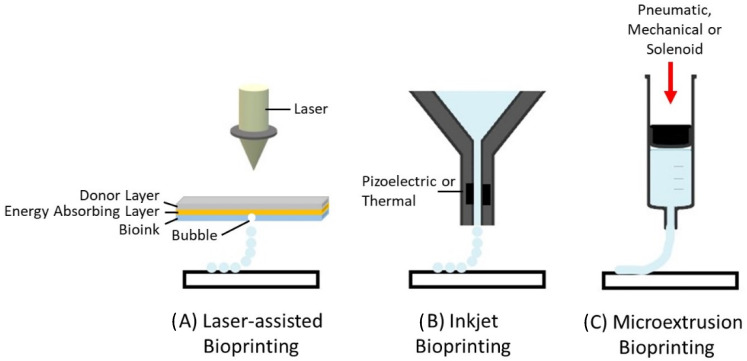
Types of 3D bioprinting. (**A**) Laser-assisted bioprinting, (**B**) inject bioprinting, (**C**) microextrusion bioprinting.

**Figure 2:**
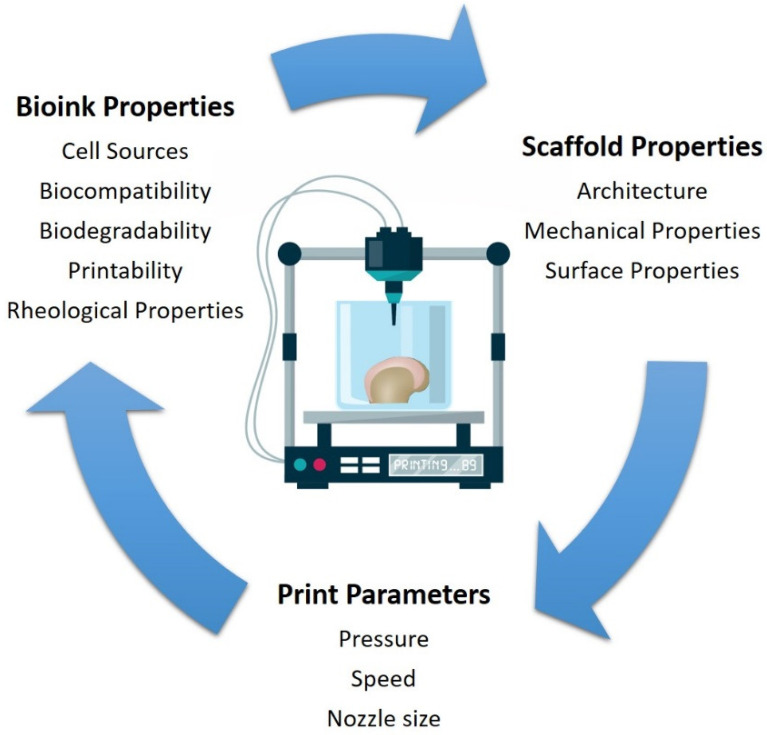
Main bioink properties, construct properties, and print parameters for extrusion-based 3D bioprinting for cartilage tissue engineering applications.

There are three main types of bioprinters currently available: (i) laser-assisted, (ii) inkjet, and (iii) microextrusion bioprinters (Figure 1). Laser-assisted bioprinters use lasers as the energy source to deposit biomaterials onto a substrate, employing the fundamentals of laser-induced forward energy [18]. Laser-assisted bioprinters can achieve very high resolutions from the picometer to the micrometer size range. They can print with a high degree of precision and can print a high cell density (~108 cells/mL) [19]. However, it has disadvantages as it is a high-cost and time-consuming process. Inkjet-based and extrusion-based bioprinting techniques are the most commonly used for tissue engineering applications. Both techniques have been successfully used for cartilage tissue engineering applications [20,21,22]. The inkjet-based method involves the secretion of droplets of bioink in liquid form, formed by piezoelectric or thermal actuation, in a controlled volume through a microfluidic reservoir to an output nozzle. The droplets can be solidified layer-by-layer to produce precise complex structures [23]. While this is a high speed, low-cost bioprinting technique, limitations include variations in droplet size and the frequent clogging of the nozzle in addition to the risk of exposing cells to high thermal and mechanical stress, and unreliable cell encapsulation [24]. Microextrusion printers extrude bioinks using a pressure gradient which can be achieved through pneumatic, mechanical, or solenoid actuation [25]. This approach is more suitable for cells and bioactive agent incorporation because it does not involve any temperature changes that could harm biological agents. It also tends to result in improved structural integrity due to the continuous and precise deposition of filaments rather than liquid droplets, however, the resolution tends to be lower than for other bioprinting techniques, in the order of 200 µm [26]. Bioinks with a wide range of rheological properties can be successfully printed using the technique. In addition, bioinks containing high volumes of cells can be successfully printed. The development of the ideal 3D bioprinted construct for cartilage tissue engineering applications using the extrusion based bioprinting process is dependent on the bioprinting parameters, ink properties, and properties relating to the construct design (Figure 2). These parameters are discussed in greater detail within this review article.

### 3.2. Bioinks for 3D Bioprinting of Cartilage Tissue Engineered Constructs 

Bioinks consist of a combination of biomaterials and cells. Bioinks must have good printability, and enable the fabrication of constructs with the appropriate mechanical strength for their intended environment whilst facilitating cell growth and proliferation. Bioactive molecules such as growth factors and signalling molecules can be incorporated into bioinks to enhance their chondrogenic properties. An extensive range of properties must therefore be considered in order to select the ideal bioink. 

#### 3.2.1. Cell Sources

Chondrocytes, as the primary cells present in cartilage tissue, are the most desirable and most predominately used cell type in the development of bioinks for cartilage tissue engineering applications. Chondrocytes can be harvested from articular cartilage and expanded to give sufficient cell numbers for use in tissue engineering applications. They have been successfully employed in the fabrication of 3D bioprinted constructs in a number of studies [27]. However, due to issues such as donor site morbidity, limited cell availability, and the cost of in vitro cell expansion, cells from alternative sources have also been investigated for bioprinting applications. These include human-derived induced pluripotent stem cells (iPSCs) [28,29] and mesenchymal stem cells harvested from the bone marrow (BMMSCs) [30,31,32], the infrapatellar fat pad (IFPMSCs), adipose tissue (ADMSCs) [33,34], synovium (sMSCs) [35], and human embryonic stem cell-derived MSCs (hESCMSCs) [29]. These stem cells can be differentiated down a chondrogenic lineage through the application of specific growth factors. One challenge relating to the use of stem cells for cartilage tissue engineering applications is their tendency to undergo hypertrophic differentiation, although recent reports suggest that sMSCs and IFPMSCs exhibit a reduced hypertrophic differentiation potential than other MSC sources, and thus may provide a preferable cell source for cartilage tissue engineering applications [36,37]. To date, an optimal stem cell source for 3D bioprinting applications has yet to be determined and further in vitro and in vivo analysis and clinical trials are required. More recent investigations have explored the use of co-cultures of two or more cell types to achieve enhanced chondrogenesis within 3D bioprinted constructs. Daly et al. developed a biofabrication strategy that enabled the engineering of structurally organised tissues by guiding the growth of cellular spheroids consisting of MSCs and chondrocytes within arrays of 3D printed polymeric microchambers [38]. Levato et al. created a zonal-like model of the articular cartilage using chondroprogenitor cells (ACPCs), BMMSCs, and chondrocytes [39]. Grogan et al. fabricated bioprinted constructs containing hESCMSCs and IFPMSCs and demonstrated their ability to promote chondrogenic neotissue as early as 2 weeks post implantation in a rabbit subchondral defect model [29]. The use of co-cultures has the potential to enhance the chondrogenic properties of the construct while offering a more cost effective and clinically applicable cell seeding approach by reducing the requirement for the in vitro expansion of cells. 

#### 3.2.2. Biocompatibility 

Biocompatibility is the compatibility of a material with living tissue. This is a key requirement for bioinks to ensure that they can promote tissue repair without causing adverse effects upon implantation in a cartilage defect site. Bioinks must be non-toxic to maintain cell viability during the 3D bioprinting process and to support the necessary cellular activity including cell adhesion, proliferation, and differentiation within the bioprinted construct without eliciting any adverse reaction. They must also be non-immunogenic and non-carcinogenic. Once implanted into the body, any negative inflammatory response or foreign body reaction to the construct will negatively impact tissue healing and may eventually lead to failure of regeneration.

#### 3.2.3. Biodegradability 

Bioprinted constructs are intended to be implanted in the body during the early stages of tissue regeneration and degrade as the body’s cells replace them, to form the desired new tissue. The bioink used for the fabrication of 3D bioprinted constructs must therefore be biodegradable. The influence of any applied crosslinking methods on degradation rates must also be considered. The rate of construct degradation must be carefully controlled to match the rate of tissue regeneration, as rapid degradation can affect the mechanical properties of the construct leading to failure of the implant [40]. The degradation of constructs can occur by physical, chemical, and/or biological processes. The fundamental modes of degradation are hydrolytic degradation, enzymatic degradation, and stimuli-associated degradation [41]. Construct degradation may elicit an immunogenic reaction, cause environmental changes, or influence cellular activity. It is therefore important that the by-products of the biodegradation process are biocompatible and non-toxic in order to be excreted from the body without negatively impacting the newly formed repair tissue or other bodily tissues or organs [42].

#### 3.2.4. Bioactivity 

Bioactivity refers to the ability of the construct to interact with its surrounding tissues and organs [43]. Bioinks used for construct fabrication must be able to interact with their environment to promote the desired cellular activity necessary for tissue regeneration whilst avoiding any undesired reactions. In the first instance, cells must be able to attach to the material surface. While naturally-derived biomaterials have intrinsic cell binding sites, synthetic materials often require surface modifications to enable cell attachment to occur. Modifications include the incorporation of cell binding peptides such as arginylglycylaspartic acid (RGD) peptides or natural biomaterials into the bioink to provide the required binding sites for cell attachment [44]. In addition to enabling cell attachment, the ideal bioinks for cartilage tissue engineering applications should ideally promote chondrogenesis within the biological environment. Various bioactive molecules have been incorporated into bioinks to enhance their chondrogenic properties. For example, growth factors from the transforming growth factor (TGF) and bone morphogenic protein (BMP) families, including TGF-β1, TGF-β3, BMP-4, and BMP-6 have been successfully incorporated into bioinks to enhance the chondrogenic properties of 3D bioprinted constructs [45,46,47]. Zhu et al. report the development of a gelatin methacrylate (GelMA)/polyethyleneglycol diacrylate (PEGDA) 3D bioprinted construct containing TGF-β1 embedded nanospheres for cartilage tissue engineering applications [45]. Wang et al. demonstrated that incorporating TGF-β3 into alginate-GelMA bioprinted constructs enhanced their chondrogenic properties [46]. Sun et al. developed 3D bioprinted gradient-structured MSC-laden constructs capable of the controlled release of TGF-β3 and BMP-4 and demonstrated their potential to support cartilage repair in vivo in a rabbit model [47].

#### 3.2.5. Printability 

The printability of a bioink, i.e., its ability to be extruded through the 3D printer in a controlled manner, is an important consideration when designing bioinks for extrusion-based bioprinting processes. The printability of a bioink is strongly dependent on a number of its other properties such as bioink homogeneity, rheological properties, viscosity, crosslinking ability, surface tension, and the bioprinting technique used [7]. The ability to print constructs with high shape fidelity is an important measure of bioink printability. This can be determined by assessing the level of structural differences between the construct design and the actual printed construct. The higher the fidelity, the less the variation between the design and printed models. In extrusion-based bioprinting, the bioprinting resolution is largely influenced by the diameter and shape of the nozzle tip. Decreasing the nozzle diameter increases the resolution, but also leads to an increase in the required extrusion force and shear stress. While the optimal needle diameter and nozzle shape have yet to be identified for chondrogenic cell populations, researchers have explored the impact of these parameters on other cell types. In general, as the shear stress increases, cell viability drops due to mechanical damage during the extrusion process. Billiet et al. reported higher viability of the hepatocarcinoma cell line (HepG2) cells when printing with conical needles rather than cylindrical needles, with 97% cell viability when printing with a dispensing pressure of ≤1 kPa and a conical needle (∅ = 200 μm) [48]. Li et al. compared the influence of needle shape on bioink flow rate and cell damage using both Schwann cells and 3T3 fibroblasts [49]. They reported greater bioink flow rates under the same pressures for tapered needles compared to cylindrical needles. Lower cell damage was also reported when the needle diameter was increased and when printing with tapered needles. At a flow rate of 0.015 mL/s and a needle diameter of 0.25 mm, cell damage remained below 5% for tapered needles for both Schwann cells and 3T3 cells, whereas cylindrical needles showed cell death of up to 20% for Schwann cells and 25% for 3T3 cells.

#### 3.2.6. Rheological Properties 

The rheological properties of bioinks play an important role in the biofabrication of constructs; influencing the ability of the bioink to deform and flow during the printing process, produce precisely controlled construct geometries, the ability of printed constructs to retain their shape after deposition, and also the cell viability during the printing process [49]. During extrusion-based bioprinting, pressure is applied to achieve extrusion of the bioink and this leads to shear stress within the bioink. Increasing shear stress leads to an exponential increase in cell damage/death and thus negatively impacts cell viability [26]. The maximum shear stress is encountered near the wall of the nozzle leading to greater cell deformation in this region. The nozzle tip diameter will also influence cell viability. Nair et al. reported that for a nozzle size of 250 µm the cell viability was reduced to less than 50% when the shear stress increased to above 150 kPa [50]. Important rheological properties to consider are viscosity and shear thinning [51].

##### Viscosity

Viscosity is a measure of a fluid’s resistance to flow. It has a substantial impact on bioprinted constructs as it can be directly linked to their mechanical properties—higher viscosity bioinks can overcome surface-tension-driven droplet formation and thus achieve the printing of continuous strands of bioink. Higher viscosity bioinks also typically result in constructs with greater mechanical properties and resistance to deformation [44]. However, high viscosity is also linked to poor cell viability and functionality, as well as the need for higher printing pressures and the printing of less accurate constructs. Conversely, low viscosity will result in the construct losing its shape, thus significantly impacting the print resolution. A balance is therefore required. Extrusion-based bioprinting has a much larger working range for viscosity than other techniques. He et al. reported good printability for sodium alginate-based bioinks with viscosity values of between 0.3 Pa·s and 30 Pa·s [52]. Bioinks with viscosities higher than this range may require significant extrusion pressure to be printed. Zhao et al. reported that the highest print fidelity was achieved for bioinks with a storage modulus, i.e., the elastic portion of the viscoelastic behaviour of a material, of between 150 and 380 Pa [53]. In order to reduce the extrusion pressures required during bioprinting, the shear-thinning properties of the bioink should be considered. The viscosity of a bioink is also dependent on the temperature at which the printing is performed, with viscosity generally increasing as the temperature decreases. 

##### Shear-Thinning 

Shear-thinning is a property of some non-Newtonian fluids, whereby the fluid viscosity decreases with increasing shear stress. This factor is important to consider as it implies that the bioink viscosity can be reduced by applying shear stress, thus allowing the smooth flow of bioink through the printer nozzle. Once deposited, the bioink will retain its original viscosity, preventing the construct from collapsing and resulting in a high printing fidelity. Shear-thinning bioinks, therefore, have improved printability while also supporting cell viability during printing. During shear-thinning, the polymer or proteins within the bioink align and disentangle at higher rates and therefore require a lower extrusion force for printing. Some biomaterials such as alginate have innate shear thinning properties. The addition of polymers, such as poloxamer 407, gellan gum, and gelMA to bioinks have also been shown to increase the shear-thinning abilities of the bioink [54]. Overall, the ideal rheological behaviour of a bioink designed for extrusion-based bioprinting should: (1) display gel behaviour given by the dominance of elasticity over viscous behaviour prior to dispensing, (2) show predominantly viscous behaviour over elastic behaviour during flow through the printing nozzle, and (3) return as closely as possible to the original gel state immediately after deposition [55].

### 3.3. Biomaterials Used in Bioinks for Cartilage Tissue Engineering Applications 

Hydrogels are hydrophilic 3D crosslinked polymeric networks that hold up to 90% water while maintaining their structure [56]. Due to their biological properties and structural similarities with native cartilage, they are considered an ideal choice as bioink materials for extrusion-based bioprinting for cartilage tissue engineering applications [57,58]. Bioinks can be fabricated using natural or synthetic materials, depending on their intended application [59]. Natural materials are those derived from natural sources, whereas synthetic materials are chemically fabricated to create custom materials with specific properties. Bioinks containing natural biomaterials are preferred by the body as they are biocompatible, biodegradable, mimic the ECM, and provide binding sites that allow cell attachment, but they can pose challenges as their properties can vary widely. Synthetic materials are more difficult to incorporate into the body as they tend to have less favourable biocompatibility and an inability to interact with cells, but have the ability to be altered to achieve the required rheological properties, have good mechanical stability, and can be altered in terms of their pH and temperature response [60,61,62]. There is a growing need for the development of new bioinks that have adequate bioprinting parameters, as well as the required material properties, including bioactivity, and physicochemical and mechanical properties [63]. This section presents an overview of the different bioinks used for cartilage tissue engineering applications, including both natural and synthetic polymer bioinks used either alone or combined (Table 1). 

#### 3.3.1. Natural Biomaterial-Based Bioinks

Natural materials used for the fabrication of bioinks for cartilage tissue engineering applications include hyaluronic acid, collagen, agarose, alginate, and gelatin. Many studies have combined one or more natural hydrogels to optimise the bioink properties [64,65,66]. In addition, the constructs fabricated from these natural hydrogels are often crosslinked using physical or chemical agents such as sodium chloride (NaCl) to improve their mechanical strength [67,68,69]. 

##### Alginate

Alginate is a biodegradable natural polymer derived from the cell walls of brown algae (Phaeophyceae). It is an ionic polysaccharide and has been investigated widely for cartilage regeneration applications due to its non-immunogenicity, non-toxicity, and good printability [70]. Alginate has been shown to integrate well with cartilage tissue and chondrocytes incorporated into alginate hydrogels have shown favourable viability [71]. It is composed of (1–4)-linked β-D-mannuronic (M) and α-L-guluronic acids (G) and contains small capillary structures that allow nutrients and water to diffuse through the material through microfluidic channels. The viscosity of alginate-based bioinks depends on the alginate concentration used, the molecular weight of the alginate used (length of the alginate chains), and the cell density and phenotype of the cells incorporated within it [72]. In terms of printability, alginate is used extensively due to its fast gelation process which can be easily induced using calcium or barium ions. It also exhibits shear-thinning properties which protect cell viability during the printing process. Jia et al. explored the influence of the material properties of alginate solutions on their printability. The study showed that the ideal density to maintain a homogenous suspension of human adipose-derived stem cells (hADSC) during the printing process was 1.05 g/mL and the ideal viscosity was between 400 mm^2^/s and 3000 mm^2^/s [73]. The viability of printed human hADSCs was >90% directly after printing and this was maintained in cell culture at 8 days post-printing. hADSCs bioprinted in alginates with viscosity values of higher than 3000 mm^2^/s showed cell viability of <90% directly after printing with 0% viable cells present following 8 days in cell culture. Despite the many favourable properties of alginate-based bioinks, disadvantages include slow and difficult to control degradation rates, poor mechanical properties, and a lack of chondroinductive properties [72]. Thus, alginate is frequently combined with additional biomaterials, such as collagen [64] and hyaluronic acid [74], and with cartilage extracellular matrix (cECM) [75] to achieve constructs with the ideal properties for cartilage tissue engineering. Rathan et al. reported that incorporating cECM into alginate-based bioinks enhanced cell viability post-printing and robust chondrogenesis in vitro [75].

##### Hyaluronic Acid

Hyaluronic acid is a polymeric glycosaminoglycan (GAG) and is one of the main constituents of articular cartilage, providing viscoelasticity and lubrication within the joint [74]. It is a critical component of synovial fluid, responsible for maintaining joint homeostasis. Its ability to enhance cartilage formation is well documented [74,76]. Hyaluronic acid is a linear polysaccharide and is composed of disaccharide units of glucuronic acid and N-acetylglucosamine. It interacts with chondrocytes through surface receptors such as CD44 and RHAMM, and it has been widely used to stimulate chondrocyte growth for tissue engineering [74,76]. Despite its favourable biological properties, hyaluronic acid lacks the mechanical and viscoelastic properties necessary for 3D bioprinted constructs and is often modified to improve these limitations [77,78,79,80]. Hyaluronic acid-based bioinks containing alginate were successfully developed by Antich et al. to achieve bioinks with suitable printability, gelling abilities, stiffness, and degradability for the fabrication of constructs using 3D bioprinting [74]. In addition, the bioprinted constructs were shown to promote chondrogenesis in vitro, demonstrating their potential for use in cartilage tissue engineering applications. 

##### Chitosan

Chitosan is a polysaccharide derived from the outer skeleton of shellfish. It is composed of glucosamine and N-acetylglucosamine and exhibits a similar structure to the GAGs present in cartilage tissue. As a result of its superior characteristics, including biocompatibility, biodegradability, bioresorbability, intrinsic antibacterial nature, and chondroconductive and chondrointegrative properties, chitosan has been widely used in tissue engineering applications [81,82]. He et al. developed chitosan-based hydrogels modified with ethylenediaminetetraacetic acid (EDTA) and demonstrated that they had favourable viscoelastic properties for use as bioinks in extrusion-based 3D bioprinting [82]. They also demonstrated the viability of chondrocytes within the bioinks and their ability to proliferate and express chondrogenic markers. However, Sheehy et al. showed in comparative studies that alginate hydrogels can promote and maintain a better chondrogenic phenotype in mesenchymal stem cells (MSCs) compared to chitosan [83].

##### Agarose

Agarose, a polysaccharide, is biocompatible with thermoreversible properties. Agarose hydrogels have been used for maintaining long-term chondrocyte cultures due to their biocompatibility, stability, self-gelling properties, non-immunogenic properties, and ability to provide a similar environment to native ECM due to its high water content [84,85,86]. Lopez-Marcial et al. reported the successful use of alginate-based bioinks for the extrusion-based bioprinting of high shape fidelity structures for engineering complex cartilaginous tissues without the requirement for additional cross-linking steps or the use of sacrificial materials [84]. Additionally, they reported that the addition of alginate to the agarose gels resulted in improved shear-thinning properties, yield strength, and print-shape fidelity than agarose alone gels. The optimal print properties, cell viability, and sGAG production were achieved using the 5% agarose-alginate-based bioinks [84]. 

##### Collagen

Collagen is the main structural protein found in cartilage and is therefore widely used as a biomaterial for cartilage tissue engineering applications [87]. Collagen is a natural polymer found abundantly in the extracellular matrix (ECM). It exhibits excellent biological properties and does not elicit an immune response [88]. The exact structure of collagen is dependent on the type, the most common being type I, type II, and type III. While the collagen in cartilage ECM is type II collagen, the majority of bioinks are produced from type I collagen as it is more readily available than type II collagen. Under physiological conditions (neutral pH and 37 °C), collagen molecules start to self-organise into fibrils forming a hydrogel [87]. The low mechanical properties of collagen bioinks and their low viscosity poses some limitations for 3D printing. For this reason, it is frequently combined with other materials to improve its properties. Alternatively, supportive hydrogels can be used when 3D bioprinting collagen-based bioinks. One example is the FRESH (freeform reversible embedding of suspended hydrogels) technique, where the printing process occurs within a secondary hydrogel, such as a gelatin slurry, which acts as a temporary thermoreversible support [89]. This approach enables the fabrication of collagen constructs with improved print fidelity and more complex shapes.

##### Gelatin

Gelatin, as a hydrolysed form of collagen, displays similar biological properties to those of collagen and is widely used for tissue engineering applications [90,91]. It has also been extensively used for other medical purposes, especially for drug capsules [92]. Gelatin is biocompatible, non-cytotoxic, water-soluble, biodegradable, promotes cell adhesion, and has low antigenicity [93]. It also contains RGD peptide binding sites which enhance cell adhesion, proliferation, and differentiation, and a matrix metalloproteinase (MMP) degradation sequence which promotes cell enzymatic degradation. Nonetheless, gelatin hydrogel alone cannot efficiently serve for cartilage regeneration because of its poor mechanical properties. It is therefore often combined with other biomaterials to produce a suitable bioink for cartilage tissue engineering applications. One such example is GelMA (gelatin methacryloyl) which is produced through the reaction of gelatin with methacrylic anhydride (MA). GelMA undergoes photoinitiated radical polymerization to form a covalently crosslinked hydrogel. GelMA hydrogels containing equine chondrocytes have been successfully used as bioinks for cartilage tissue engineering, achieving high levels of cell viability and production of aggrecan and collagen type II following 4 weeks in vitro culture [94].

#### 3.3.2. Synthetic Biomaterial-Based Bioinks

Numerous synthetic polymers are used for cartilage tissue engineering, including poloxamers, polycaprolactone (PCL), poly-lactic acid (PLA), and poly-glycolic-acid (PGA). These polymers have been combined with other synthetic biomaterials and with natural biomaterials in order to improve properties such as mechanical properties, crosslinking, and printability for use in cartilage tissue engineering applications and to stimulate chondrogenesis [95,96,97]. Synthetic polymers are also used as sacrificial bioinks that support the construct structure during the bioprinting process. Poloaxmers are particularly suited for use as sacrificial polymers due to their thermoreversible gelation properties. For example, Pluronic^®^ (a commercially available poloxamer, Sigma Aldrich, Wicklow, Ireland) is liquid at <4 °C and forms a gel at >16 °C [98]. PCL is also frequently used to improve the mechanical properties of bioprinted constructs. It can be easily blended with other polymers. Jung et al. fabricated a cartilage extracellular matrix (CAM)/silk fibroin construct co-printed with polycaprolactone (PCL) as a framework to enhance the structural stability of the printed construct [99]. Mouser et al. developed bioinks containing methacrylated hyaluronic acid (HAMA) added to thermosensitive hydrogels composed of methacrylated poly[N-(2-hydroxypropyl)methacrylamide mono/dilactate] (pHPMA-lac)/polyethylene glycol (PEG) triblock copolymers and co-printed them with PCL to generate porous or solid constructs with different mesh sizes [100]. They achieved constructs with a Young’s modulus in the range of native cartilage (3.5–4.6 MPa).

PEG is also widely used in cartilage tissue engineering applications due to its ability to finely tune its properties to meet particular requirements. For example, the methacrylation of PEG can achieve photocrosslinkable PEG dimethacrylate (PEGDA) and poly(ethylene glycol) dimethacrylate (PEGDMA). These materials are also widely used in drug delivery applications for the controlled release of hydrophobic drugs. Chen et al. developed a structure for supporting hydrogel bioink, containing aldehyde hyaluronic acid, N-carboxymethyl chitosan, gelatin, and PEG succinimidyl glurate [101]. They demonstrated that this bioink enabled the printing of constructs with viscoelastic properties and self-healing behaviour with the potential for use in cartilage tissue engineering applications. 

While the use of synthetic polymers has been shown to enhance the bioink printability and the stability and mechanical properties of 3D bioprinted constructs, they have been shown to be less favourable in terms of promoting chondrogenesis. Daly et al. compared BMMSC chondrogenesis in bioprinted constructs composed of agarose, alginate, GelMA, and PEGMA-based bioinks, reporting that agarose and alginate were supportive of hyaline-like cartilage tissue formation, with type II collagen deposition, whereas GelMA and PEGMA-based bioinks resulted in the formation of fibrocartilage, typically composed of collagen type I [32].

### 3.4. 3D Bioprinted Constructs for Cartilage Tissue Engineering

Three-dimensional bioprinted constructs aim to provide a microenvironment using a combination of cells, growth factors, and biomaterials in which cells can grow and proliferate into distinct tissues. An ideal construct should simulate the mechanical and biological properties of the native ECM of the desired tissue. The ECM is the non-cellular component of tissues and organs, providing cell adhesion, mechanical support to the cellular constituents, and initiating biochemical reactions for tissue morphogenesis, differentiation, and homeostasis [109]. Each tissue has a unique ECM, differentiated by its physical, chemical, and topological compositions. In 3D bioprinting, the bioprinter controls the deposition of the bioink to determine the shape and structure of the construct. Key properties of 3D bioprinted constructs include construct architecture, construct mechanical properties, and the surface properties of the construct. 

#### 3.4.1. Fabrication of 3D Bioprinted Constructs

The process of construct fabrication using 3D bioprinting first involves designing the construct, followed by the printing of the construct using the 3D bioprinter. Constructs are typically designed using computer-aided design (CAD) software. These CAD files are then converted to G-code, a programming language that communicates with the 3D printer to instruct it on how to print the construct by indicating the printing parameters and pathway. Following the printing of the construct, various post-processing procedures can be applied including physical or chemical crosslinking to solidify the construct, ensuring it maintains its geometric structure. Crosslinking is a critical element in 3D bioprinting as it strongly influences the eventual mechanical and physiochemical characteristics of bioprinted constructs and impacts the cellular behaviour of the incorporated cells [110].

#### 3.4.2. Architecture of 3D Bioprinted Constructs

Construct architecture refers to the overall geometry of a construct and its internal microarchitecture. The porosity, pore shape, and pore size are critical microarchitectural parameters to consider in the fabrication of constructs. Porosity is the measure of void spaces within a structure and has a direct correlation with the construct mechanical and biological properties. Open, interconnected pores facilitate the diffusion of nutrients and other small molecules through the construct to stimulate cell growth, vascularisation, and waste removal [111]. Koo et al. compared cellular activity in 3D printed porous mesh collagen constructs to non-porous collagen gels and reported high viability in the core of the porous collagen constructs and high levels of cell death in the core region of the non-porous hydrogels after 7 days in vitro culture [106]. Pore size is also an important parameter. If the pore size is too small, cell migration and diffusion of nutrients are limited. Contrarily, if the pores are too big, a decrease in surface area results, limiting the ability of cells to adhere to the constructs. A compromise in the selection of pore size, therefore, needs to be established in the design of bioprinted constructs. Numerous studies have investigated the optimal pore size of constructs for cartilage tissue engineering applications. Zhang et al. reported an ideal pore size range for collagen-based constructs for cartilage tissue engineering of 150–250 μm [112] and Matsiko et al. reported an optimal mean pore size of 300 μm [113]. The pore geometry has also been shown to influence cellular response. Ferlin et al. explored the influence of 3D printed porous architecture on MSC differentiation, demonstrating that constructs fabricated with ordered cubic pores significantly increase the gene expression of MSCs undergoing chondrogenesis when compared to constructs with ordered cylindrical pores. [114]. Soufivand et al. compared the mechanical properties of PCL-based constructs printed with lattice, wavy, hexagonal, and shifted microstructures [115]. They reported that the compressive elastic moduli of the constructs varied from 1.6 MPa to 56.7 MPa depending on the construct microstructure. Thus, tailoring of the construct microstructure is important in order to achieve constructs with the ideal mechanical properties for cartilage tissue engineering applications. Gaetani et al. reported that lattice structures support higher cell viability and proliferation rate because they offer a conducive environment for nutrient supply and waste excretion [116]. The strut diameter of 3D bioprinted constructs is dependent on the bioprinting parameters such as the plotting speed, dispensing inlet pressure, temperature, and needle internal diameter. Billiet et al. demonstrated that for extrusion-based bioprinted GelMA constructs, strut diameters of between 150 µm and 2000 µm could be achieved by varying the following print parameters: plotting speed (100–1000 mm/min), dispensing inlet pressure (100–500 kPa), temperature (24.5–27.5 °C), and needle internal diameter (150–200 µm) [48].

More recent developments in the 3D bioprinting of constructs for cartilage tissue engineering have focused on the fabrication of constructs that mimic the zonal structure of cartilage tissue. Constructs with gradient physical and mechanical properties and chemical and biological compositions have been developed [39,47,117,118,119,120]. Dimaraki et al. developed alginate-based bioprinted constructs with gradient cell densities designed to replicate the differing cell densities within each zone of the articular cartilage [117]. Levato et al. reported the 3D printing of constructs using three different materials loaded in multi-dispenser heads: (1) a superficial zone-mimicking bioink consisting of an articular cartilage-resident chondroprogenitor cell (ACPC)-laden GelMA, (2) a middle/deep zone-mimicking bioink composed of bone marrow mesenchymal stromal cell (MSC)-laden GelMA, and (3) Pluronic F-127 as a sacrificial ink to support (MSC)-laden GelMA during the process [39]. Sun et al. successfully 3D printed dual-factor releasing MSC-laden gradient constructs for cartilage repair applications [47]. Within the study, bone morphogenetic protein 4 (BMP4) and transforming growth factor–β3 (TGFβ3) were encapsulated within PLGA microspheres and incorporated into the hydrogel-based bioinks prior to printing in a layered fashion to achieve spatiotemporal growth factor release within the defect site. In vitro assessment demonstrated the presence of abundant cartilaginous matrix containing collagen type II and aggrecan in a gradient manner primarily in the superficial layers with TGFβ3 delivery, whereas hypertrophic marker collagen type X was primarily expressed in the deepest zone. 

#### 3.4.3. Mechanical Properties of 3D Bioprinted Constructs

The mechanical properties of a bioprinted construct should ideally match that of the native tissue for optimum tissue regeneration [121]. The Young’s modulus of the surface region of articular cartilage is reported to be 0.28 ± 0.16 MPa and for the deep zone of articular cartilage is reported to be 0.73 ± 0.26 MPa [122]. In addition, 3D bioprinted constructs should have sufficient mechanical properties to withstand surgical handling during implantation and retain their mechanical strength post-implantation until the completion of the tissue regeneration process. The mechanical strength of a construct is influenced by the bioink composition, structural design of the construct, and the post-printing conditions, e.g., crosslinking techniques [123]. The addition of synthetic materials such as PCL and PLA to bioinks can increase the mechanical strength of 3D bioprinted constructs. 

#### 3.4.4. Surface Properties of 3D Bioprinted Constructs

Surface properties such as surface energy, chemistry, and topology are important factors to consider when designing a 3D bioprinted construct. The hydrophobicity/hydrophilicity of the outer surface of the construct is another key factor to consider. These surface properties influence the relationship between the construct and proteins in the body, which affect cell attachment, proliferation, and differentiation capabilities. For constructs that have poor surface properties, bioactive adhesive molecules such as collagen, gelatin, fibronectin, growth factors, insulin, etc., can be covalently or physically attached to the biomaterial surface. 

## 4. Clinical Translation of 3D Bioprinted Constructs for Cartilage Repair Applications

**Figure 3:**
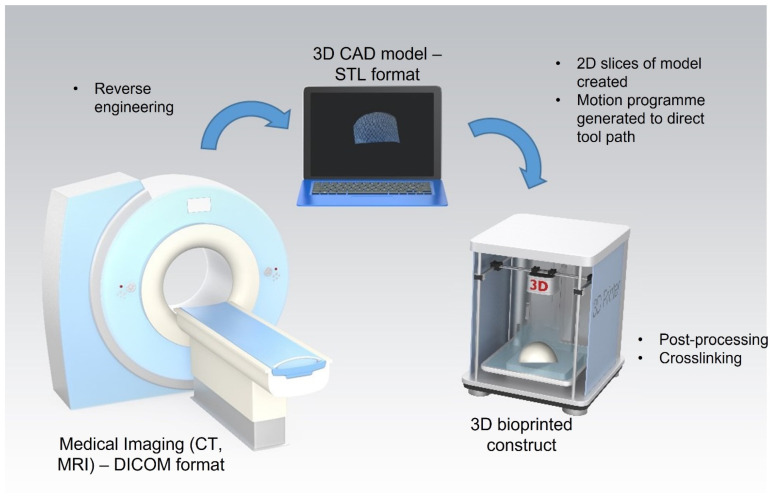
Process for 3D bioprinting of patient specific constructs for cartilage tissue engineering applications. Stage 1: 3D image of cartilage defect captured using computed tomography (CT) or magnetic resonance (MRI) and stored in the Digital Imaging and Communications in Medicine (DICOM) format. Stage 2: DICOM file is reverse-engineered and imported into computer-aided design (CAD) software and then converted into an STL file. Stage 3: A motion programme is created to provide the tool path information for the printer. The desired construct is bioprinted in a layer-by-layer fashion and post-processing/crosslinking applied.

Extrusion-based 3D bioprinting has shown promise for the fabrication of constructs composed of both natural and synthetic biomaterial-based bioinks for cartilage tissue engineering applications. While the ability of these constructs to promote chondrogenesis has been demonstrated in vitro, further pre-clinical studies are required to demonstrate their efficacy in vivo. To date, 3D bioprinted constructs have yet to be successfully translated to the clinic. The technique has been shown to have good reproducibility and potential for mass scalability and it also shows promise for use in personalised medicine. However, limitations remain including high costs and complex regulatory pathways for the approval of tissued engineered constructs. The proposed clinical application of this technique in a personalised medicine approach involves three stages: (i) medical imagery, (ii) construct design, and (iii) construct bioprinting (Figure 3). The medical imaging stage employs imaging techniques such as computed tomography (CT) and magnetic resonance (MRI) to obtain a 3D image of the cartilage defect. This data is stored in the Digital Imaging and Communications in Medicine (DICOM) format, the standard image file format for medical imaging. Following this, the DICOM file is reverse-engineered and imported into computer-aided design (CAD) software. This enables the generation of a surface model that mimics the shape and structure of the defect site. This model is converted into an STL file and then used to create two-dimensional (2D) slices of the construct. A motion programme is then created which contains codes that provide the tool path information for the printer. Patient cells would then be harvested and combined with the desired biomaterial to produce a bioink. The desired construct would then be bioprinted in a layer-by-layer fashion. Finally, any post-processing or crosslinking required would be applied to achieve a final 3D bioprinted construct ready for implantation into the defect site [124]. 

An alternative approach is the use of in situ bioprinting where bioinks are directly printed into the defect site by the surgeon within a clinical setting. This removes the requirement for the bioprinted construct to be handled by the surgeon prior to implantation. This approach may provide particular advantages for the reconstruction of complex geometries, such as curved surfaces [125]. An interesting example of this approach is the BioPen (Australian Research Council Centre of Excellence for Electromaterials Sciences (ACES), University of Wollongong (UOW)), a handheld, 3D bioprinting device dedicated to in situ 3D bioprinting for cartilage tissue repair [126]. This device is a handheld co-axial extrusion device that allows the deposition of cells embedded in a hydrogel material in the surgical setting. The complex regulatory pathway for tissue-engineered constructs presents a major challenge to the successful translation of 3D bioprinting technologies to the clinic. Further research is required to ensure that bioprinted products are reproducible, high quality, safe, and effective at achieving repair of cartilage tissue [127]. Obtaining ethical approval for the harvest and expansion of stem cells in the laboratory and subsequently, their use in surgery presents a challenge to clinical translation. As a relatively new technique, there is a lack of bioprinting-specific standards and this poses further challenges when obtaining regulatory approval for bioprinted constructs. In order to overcome these challenges, close collaboration between academia, industry, and regulators will be essential.

## 5. Conclusions and Future Perspectives

While 3D bioprinting is still in the early stages of development, with remaining clinical, economic, and ethical challenges, it has the potential to greatly impact the clinical treatment approaches for cartilage injuries, with the promise of achieving rapid, long-lasting regeneration of cartilage tissue damage. In particular, further in vitro and in vivo assessments of 3D bioprinted constructs is required in order to determine the optimal bioinks and 3D bioprinting parameters required to achieve 3D bioprinted constructs capable of promoting cartilage tissue regeneration. Three-dimensional bioprinting has shown the potential to produce mechanically viable bioprinted constructs capable of cell growth and proliferation, however, challenges such as biocompatibility and printability must be overcome before 3D bioprinting becomes clinically relevant. Furthermore, as tissue-engineering approaches advance toward clinical applications, there is a growing need for the development of 3D bioprinted constructs that more closely recapitulate the native mechanical strength, collagen architecture, surface contour, geometry, and morphology of the native joint.

The emergence of four-dimensional (4D) bioprinting approaches, where the transformation of properties and physical, chemical, and biological compositions of 3D constructs occur over time, will likely bring important advances for cartilage tissue engineering applications. These time-dependent changes will enable the development of constructs that can adapt to stimuli from the environment such as humidity, temperature, and chemicals. These approaches would enable greater control over construct properties and allow greater control over the delivery of drugs and growth factors from 3D bioprinted constructs. 

The application of artificial intelligence (AI) and machine learning (ML) in the optimisation of the 3D bioprinting process has the potential to enhance the rate of development in this area, resulting in the delivery of 3D bioprinted constructs to the market more rapidly [128]. Ruberu et al. successfully applied machine learning as a novel tool to evaluate printability quantitatively and to fast track optimisation of extrusion-based bioprinting in achieving a reproducible 3D construct [129]. Some challenges in relation to the application of these AI and ML techniques to the bioprinting process remain, including the lack of training databases to train new AI and/or ML algorithms. The development of a ‘digital twin’ of the articular cartilage that would enable the virtual assessment of new 3D bioprinted materials, and reduce the requirement for costly and time-consuming physical experimentation would also enable significant advances in this area. 

Overall, the future of 3D bioprinting is promising and it is expected to drive major advancements both within research and the clinical environment in the future, including in areas of reconstructive surgery, medical imagery, drug development and delivery, and cancer research. Ultimately, 3D bioprinting is expected to become an essential tool in the treatment of cartilage injury and disease, and overall will improve the quality of life for patients.

## Figures and Tables

**Table 1:** Natural and synthetic biomaterials-based bioinks for cartilage tissue engineering applications.

Natural Polymers Bioinks				
Bioink Polymers	Cell Viability	Crosslinker	Outcomes	Ref.
Alginate	Chondrocytes: above 70% after 24 h of incubation	CaCl_2_	The addition of HA on the NC-Alg based bioink resulted in significantly higher cell viability. Improvement of rheological properties.	[102]
Hyaluronic acid	Human articular chondrocytes: 85%	CaCl_2_	Provided suitable mechanical properties. Creation of a proper biomimetic hybrid construct.	[74]
Gelatin	Human umbilical cord blood-derived (hUCB) MSCs: 75%	Streptoverticillium mobaraense (6 h)	Strengthens the promotion of chondrogenic differentiation.	[103]
Chitosan	Rabbit chondrocytes: mesh group: (95.9 ± 1.3%); control: (96.1 ± 2.1%)	Ethylenediaminetetraacetic acid (EDTA)/ CaCl2 (30–45 min)	Fast gelation. High printing fidelity. Suitable mechanical properties and stability.	[82]
Fibrin	ATDC5 cells: higher than 90%	Photo-crosslinking with UV.	High mechanical properties. Long-term and constant rate growth factor.	[104]
Gellan gum	Rabbit chondrocytes/human placental MSCs: nearly 100%	CaCl_2_ (5 min)	Easy printing process. Maintains cell activity.	[105]
Agarose	Bovine articular chondrocytes: above ∼70% cell survival at day 28	NA	High shape fidelity. No need for additional crosslinking.	[84]
Collagen	Rabbit articular chondrocytes: 84% of cell viability	Genipin (0.5, 1, 3, 6 h)	High mechanical and cell viability.	[106]
**Synthetic Polymers Bioinks**				
**Bioink Polymers**	**Cell Viability**	**Crosslinker**	**Outcomes**	**Ref.**
PCL/Extra cellular matrix (ECM)	Human inferior turbinate-tissue derived MSCs (hTMSCs): >95% at day 1, >90% at day 7 and 14	Incubation at 37 °C temperature for 30 min	Chondrogenic differentiation of cells within the construct, with greater expression of SOX9 and type II collagen than in collagen only constructs.	[107]
PEG	Chondrocytes: 93.83 ± 2.40%	PEG-SG	High permeability. Biocompatible components. Low stiffness.	[101]
HAMA-Phpma-lac/PEG	Chondrocytes: high cell survival	UV light	Increase stiffness and concentration. Increase cartilage matrix production.	[100]
Hyaluronic acid/poly(glucidol)/PCL	Human and equine BMMSCs: high cell survival after the printing process	UV light	Suitable mechanical properties. Harmless printing process for the cells.	[108]
